# A Cross-Knotted Suture Basket Technique for Large Nonmagnetic Intraocular Foreign Body Removal

**DOI:** 10.1155/2020/1061462

**Published:** 2020-04-25

**Authors:** Jian Cao, Baihua Chen, Yun Li

**Affiliations:** ^1^Department of Ophthalmology, the 2nd Xiangya Hospital of Central South University, Changsha, Hunan, China; ^2^Hunan Clinical Research Center of Ophthalmic Disease, Changsha, Hunan, China

## Abstract

**Purpose:**

To report a novel technique of cross-knotted suture basket and to test its effectiveness in large nonmagnetic intraocular foreign body (IOFB) removal.

**Methods:**

A 7/0 Vicryl suture was cut in half and cross-knotted, and four ends were introduced into a 23G needle to form a basket. Pig eyes were used to set up the IOFB model, and the effectiveness of the suture basket in the removal of large nonmagnetic intraocular foreign bodies was tested.

**Results:**

Several modifications can be made to adapt to different situations. For the materials (stone, metal, glass, and wood) and shapes (irregular, spherical, and rectangle) of large IOFB tested, the cross-knotted suture basket successfully removed all kinds of IOFBs.

**Conclusion:**

The suture basket technique provides an accessible, safe, and effective alternative in large nonmagnetic IOFB removal. It can be adapted and interchangedand also worth's further clinical investigations.

## 1. Introduction

The estimated annual incidence of open globe injuries (OGI) is 3.5/100 000 in the industrialized world, and 17–41% of OGI has intraocular foreign bodies (IOFBs) present [1–3], as a common clinical scenario.

Patients with retained IOFBs are usually managed surgically by pars plana vitrectomy and IOFB removal, the latter as the crucial step of the surgery in most cases. Despite the vitreoretinal surgery advances, 25% of patients with IOFBs have a guarded visual prognosis [4]. Among various IOFBs, small to medium magnetic IOFB can be removed by magnets and nonmagnetic IOFBs by various intraocular forceps. However, in case of large IOFBs, especially nonmagnetic, whether removal with either magnets or forceps is impossible remains a particular challenge.

We report a novel technique of cross-knotted suture basket construction and surgical skills, which provides a controlled, comprehensively applicable way of large nonmagnetic IOFB removal.

## 2. Materials and Methods

### 2.1. Cross-Knotted Suture Basket Construction

**Figure 1:**
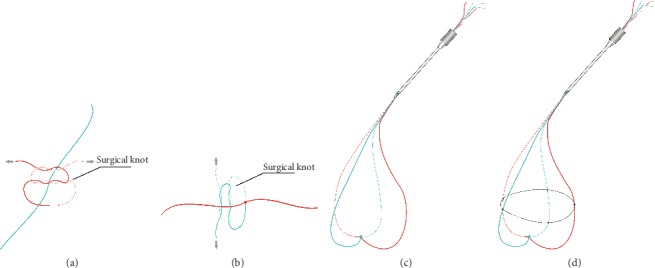
Construction of the cross-knotted suture basket. Two perpendicular sutures (in our case 7/0 Vicryl) were knotted to each other with two sets of crossed surgical knots ((a) and (b)). Then the four ends of the cross-knotted basket are all introduced into a 23- or 25-gauge needle, to form a soft basket (c), which is later to be inserted into the eye through a pars plana or limbal access with the needle.

7–0 Vicryl suture was cut in half with both needles removed. The two sutures were placed perpendicularly, and the suture underneath was tied with a surgical knot (Figure 1(a)), followed by another surgical knot of the other suture, forming a cross-knotted suture that will not easily slide (Figure 1(b)). Then the four ends of the cross-knotted basket were glued together with viscoelastic agent and all introduced into a 20- or 23-gauge needle, to form a soft basket (Figure 1(c)), which is later to be inserted into the eye through a pars plana (phakic eye) or limbal access (aphakic eye) with the needle.

For large IOFBs with a smooth spherical surface, e.g., shotgun pellets or pebbles, an extra strengthen line is tied with each of the four sutures (Figure 1(d)), forming a spider web-like basket, to avoid the IOFB's slippage out of the basket.

### 2.2. Cross-Knotted Suture Basket Wet Lab Experiment

For the ethical consideration, the feasibility of the suture basket was tested in the wet lab in a pig eye. Fresh pig eyes were obtained from a local slaughterhouse. Experiments were performed in a standard vitreoretinal operating room setting. To set up the large IOFB model, a limbal incision size coinciding with the tested foreign body was made and lensectomy and curvilinear capsulorrhexis of both anterior and posterior capsule was done to clear the visual axis. The foreign bodies were put inside the vitreous cavity via the limbal incision. Large IOFBs (defined as its smallest axis larger than the largest foreign body forceps distance) of different materials (stone, metal, glass, and wood) and shapes (irregular, spherical, and cylindrical) were tested.

### 2.3. Surgical Technique

Surgeries were performed using CONSTELLATION® Vision vitrectomy system (Alcon, USA) under ZEISS OPMI Lumera *T* surgical microscope. Surgeries were performed by one of the authors (L.Y.).

**Figure 2:**
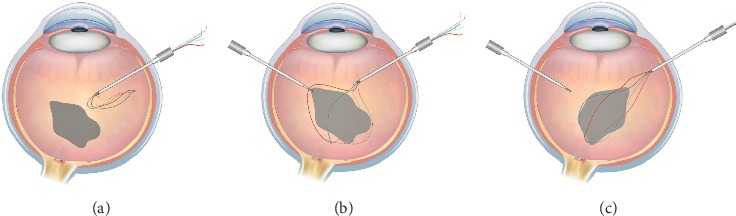
Intraocular foreign body (IOFB) removal by cross-knotted suture basket. The basket was introduced into the eye through the limbal incision or pars plana trocar. (a) Then the four suture was placed appropriately around the IOFB. (b) The IOFB position is adjusted inside the basket to make its maximal cross-section smallest to the exit plane of the IOFB (Figure 1(c)), enabling the exit to be as small as possible.

For the wet lab experiments, we began the surgery by placing 23-gauge cannulas in a routine way for a standard 23G pars plana vitrectomy, followed by a core vitrectomy, till the IOFB is fully untangled from the vitreous. The premade cross-knotted suture basket was inserted inside the eye (Figure 2(a)), another instrument (i.e., light pipe or intraocular forceps) helped to place the basket sutures around the IOFB (Figure 2(b)); when ready, retraction of the suture will constrict the basket and tighten IOFB to the needle shaft. The IOFB position in the basket can be adjusted ideally with its smallest maximal cross-section parallel to the exit plane (perpendicular to the needle shaft axis) (Figure 2(c)), so it could come out of the eye through the smallest incision. The IOFB exit could be direct or by shaking hand technique between the basket and a tweezer, whichever convenient for the surgeon.

## 3. Results

In the wet lab experiment, different materials (stone, metal, glass, and wood) and shapes (irregular, spherical, and cylindrical) of IOFB were tested, and in all cases, IOFBs were successfully removed by the cross-knotted suture basket. During the procedure, it showed great control and minimal iatrogenic injury, and procedures were recorded (see video, Supplement 1).

## 4. Discussion

Nonmagnetic IOFBs are not uncommon in OGI [1, 5]. Various surgical skills have been described in nonmagnetic IOFB removal; most frequently used instrument include intraocular foreign body forceps [6], aspiration tips [7], or perfluorocarbon liquid (PFCL) to move the nonmagnetic IOFBs outside the macula [8]. These techniques are of limited use in large rigid IOFBs, especially when it is nonmagnetic.

Various attempts of dealing with this situation include alligator forceps [9], endoscope [10], special designed forceps [11, 12], direct removal with large incision [13], and flipping the patient to a face-down position [14]. These techniques either require special instrumentation with limited access to most ophthalmologists or very traumatic with a high risk of intraoperative and postoperative complications. There is a simple but elegant design of IOFB lasso [15] or snare [16], with the limitation of monoplane holding force, which is insufficient in spherical or olive-shaped IOFB.

We hereby introduced a novel idea of knitting the IOFB removal basket with sutures. In the surgical video (see Supplement 1), we demonstrate the skill in the pig eye because of comparatively scarcity of such a large nonmagnetic foreign body patients and also ethical considerations. In the video, we can see that this basket technique can easily trap round and smooth IOFBs, as would be extremely difficult to extract by routine skills. The whole procedure is free of major complications.

This technique has several advantages. (1) It is made of two simple, inexpensive, handy materials (needle and suture), by extremely easy procedure, so the surgeon can stop at any point of the surgery to make it without the need of any preparation for instrumentations. (2) Compared to the monoplane design of the IOFB snare and lasso, the basket provides a multiplane grasp force of the IOFB, which is much easier and safer in the removal of IOFBs with larger center and smaller ends (e.g., spherical, olive, or diamond shaped IOFBs). (3) When launched properly in the basket, the IOFB could be adjusted to the position where its smallest maximal cross section is parallel to the exit plane of the IOFB, enabling the exit to be the smallest. (4) The suture basket is soft so that it could shape with IOFB of any shape and size. (5) Elements of the basket could be readily interchanged. For example, we choose 7–0 Vicryl because it is most accessible for us, provides enough strength and friction with the IOFB, and certainly could be changed with any other appropriate sutures. For more challenging cases like heavy smooth shotgun pellet, a strengthen line could be added to further stabilize the sphere, but the knitting pattern could be kaleidoscopic, according to each surgeon's own skills. As a matter of fact, its appliance is not confined to the large nonmagnetic IOFBs, for surgical units or accidental occasions with no access to various foreign body forceps, it could be a valuable alternative. To be brief, this is a preliminary method that needs, and also worth, further clinical investigations.

## Data Availability

The wet lab video of this technique used to support the findings of this study are included within the article.

## Supplementary Materials

Supplementary Materials The video recorded the procedures of how we tested the cross-knotted suture basket in the wet lab experiment. The video legend is “A suture basket is made of: 1.two sets of 3-1-1 surgical knots of perpendicular 7/0 Vicryl suture and 2. simultaneous introduction of 4 ends of the suture into a 23-gauge needle. The effectiveness of the suture basket was tested with intraocular foreign bodies (IOFBs) of different materials (stone, metal, glass, and wood) and shapes (irregular, spherical, and cylindrical). The video showed a plastic pellet ball and a large rectangle glass. “At the end of the manuscript, there was a video legend of the supplementary material.
